# Supplementary material for: Experimental Insight into the Structural and Functional Roles of the ‘Black’ and ‘Gray’ Clusters in Recoverin, a Calcium Binding Protein with Four EF-Hand Motifs
Source: Molecules. 2019 Jul 8;24(13):2494. doi: 10.3390/molecules24132494 (PMC6650976; doi:10.3390/molecules24132494)
Supplement: Supplementary file 1 [file molecules-24-02494-s001.pdf]

Supplementary materials

Experimental insight into the structural and functional roles of the 'black' and 'gray' clusters in recoverin, a calcium binding protein with four EF-hand motifs

Sergey E. Permyakov[a], Alisa S. Vologzhannikova[a], Ekaterina L. Nemashkalova[a], Alexei S. Kazakov[a,b], Alexander I. Denesyuk[a,c], Konstantin Denessiouk[c,d], Viktoriia E. Baksheeva[e], Andrey A. Zamyatnin Jr.[e,f], Evgeni Yu. Zernii[e,f], Vladimir N. Uversky[a,g,*], and Eugene A. Permyakov[a,b,*]

[a]*Institute for Biological Instrumentation of the Russian Academy of Sciences, Federal Research Center Pushchino Scientific Center for Biological Research of the Russian Academy of Sciences, Pushchino, Moscow region, 142290 Russia.*

[b]*Department of Biomedical Engineering, Pushchino State Institute of Natural Sciences, Pushchino, Moscow region, 142290 Russia.*

[c] *Structural Bioinformatics Laboratory, Biochemistry, Faculty of Science and Engineering, Åbo Akademi University, Turku 20520, Finland.*

[d] *Pharmaceutical Sciences Laboratory, Pharmacy, Faculty of Science and Engineering, Åbo Akademi University, Turku 20520, Finland.*

[e]*Belozersky Institute of Physico-Chemical Biology, Lomonosov Moscow State University, 119992 Moscow, Russia.*

[f]*Institute of Molecular Medicine, Sechenov First Moscow State Medical University, Moscow, Russia,*

[g]*Department of Molecular Medicine and USF Health Byrd Alzheimer's Research Institute, Morsani College of Medicine, University of South Florida, Tampa, Florida 33612, USA.*

Table S1. The values of ε_{280nm} for rWT human recoverin and its cluster mutants (a molar extinction coefficient of 280 nm, calculated using online software ProtParam Expasy (https://web.expasy.org/protparam/) and substitutions in the corresponding codons of synthetic oligonucleotides for each mutants of rWT human recoverin.

Protein		*Substitution*	*ε_{280}, $M^{-1}cм^{-1}$*	*Major molecular mass by the data of mass spectrometry, Da / its % from sample general quantity*
rWT			25,440	23,208 / 89
N-domain				
Black cluster	F35A	TTC by GCC	25,440	23,138 / 80
	F83A	TTC by GCC	25,440	23,134 / 100
	Y86A	TAC by GCC	23,950	23,116 / 66
Gray cluster	F70A	TTC by GCC	25,440	23,130 / 85
	Q46A	CAG by GCC	25,440	23,152 / 60
	F49A	TTC by GCC	25,440	23,138 / 100
C-domain				
Black cluster	F106A	TTC by GCC	25,440	23,132 / 83
	E169A	GAG by GCC	25,440	23,156 / 100
	F172A	TTC by GCC	25,440	23,131 / 95
Gray cluster	W156A	TGG by GCC	19,940	23,094 / 59
	K119A	AAG by GCC	25,440	23,151 / 55
	V122A	GTG by GCC	25,440	23,179 / 84

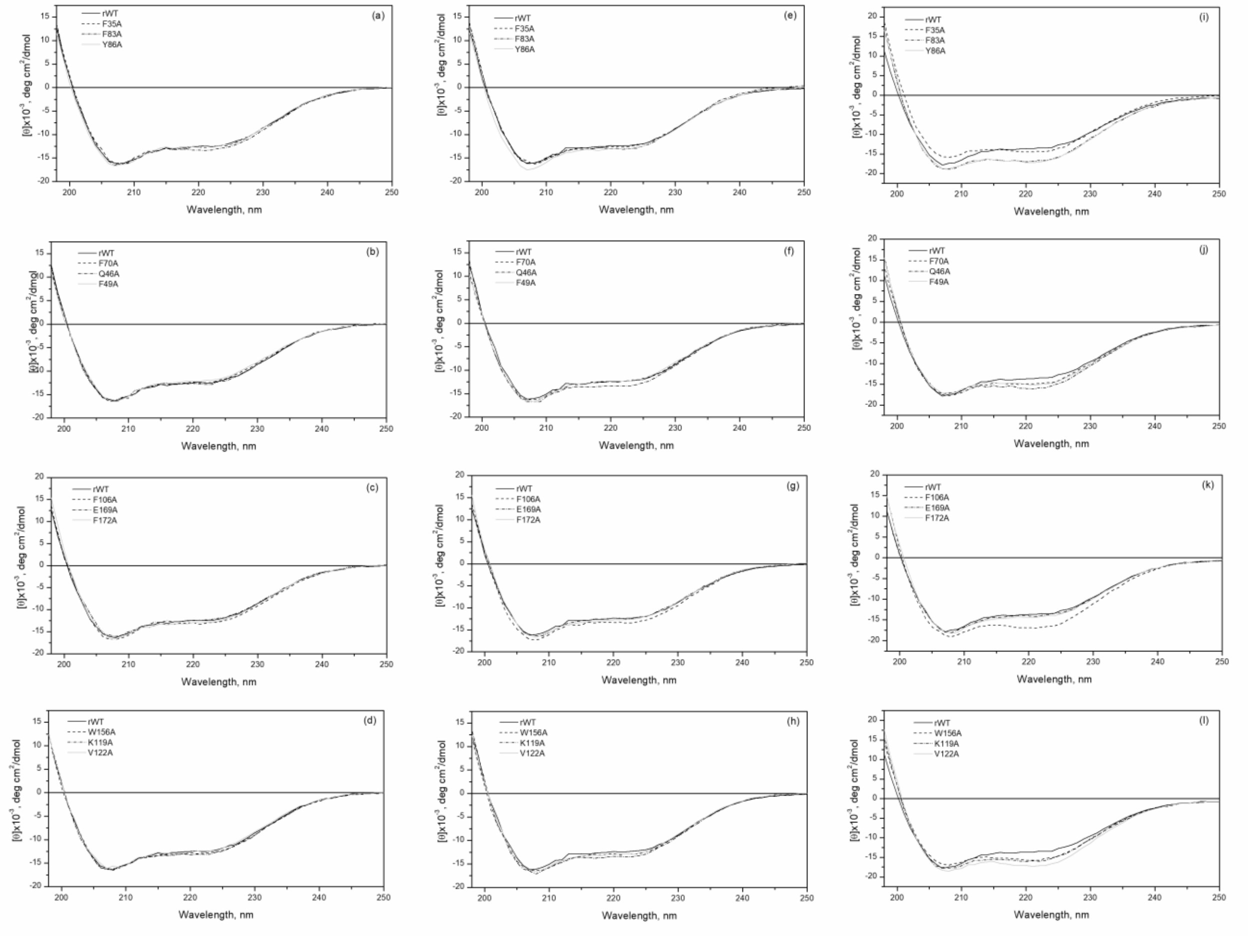

Figure S1. Far-UV CD spectra of apo (1mM EDTA) (a – 'black' cluster of the N-domain, b – 'gray' cluster of the N-domain, c – 'black' cluster of the C-domain, d – 'gray' cluster of the C-domain), Mg^{2+}- (1mM EGTA, 1mM $MgCl_2$) (e – 'black' cluster of the N-domain, f – 'gray' cluster of the N-domain, g – 'black' cluster of the C-domain, h – 'gray' cluster of the C-domain) and Ca^{2+}-loaded (1mM $CaCl_2$) (i – 'black' cluster of the N-domain, j – 'gray' cluster of the N-domain, k – 'black' cluster of the C-domain, l – 'gray' cluster of the C-domain) forms of rWT human recoverin and its cluster mutants at 15 ℃. 10 mM Tricine-KOH, 50 mM KCl, 20 μM DTT pH 7.4.

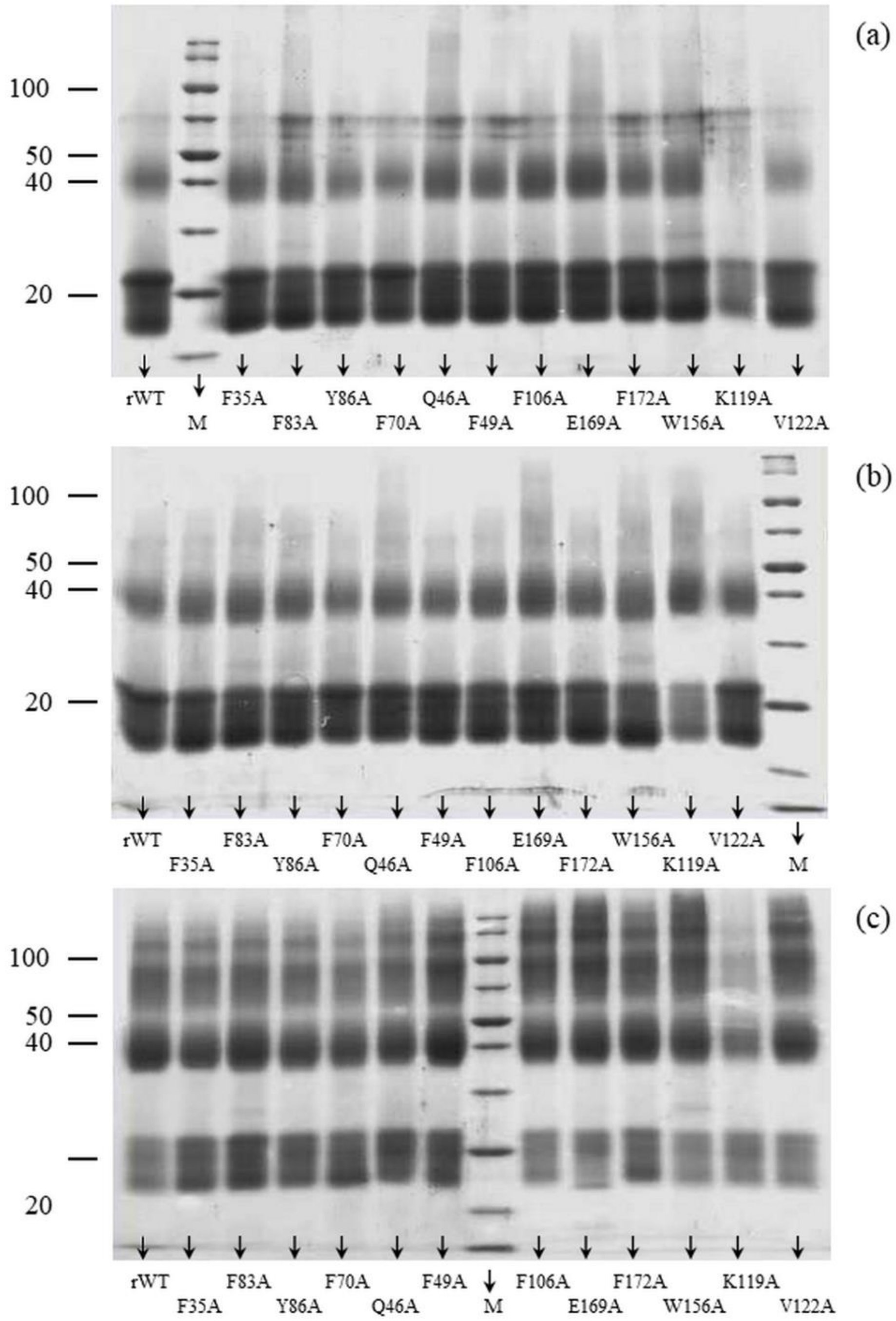

Figure S2. Distribution of aggregated forms of recombinant wild type human recoverin and its cluster mutants obtained from experiments on chemical crosslinking by glutaric aldehyde (0.02%) at 20 ℃. Protein concentration 1 mg/ml. 20 mM Tricine-KOH pH 7.4, 50 mM KCl, 1 mM DTT; 1 mM EDTA (for apo-proteins), 1mM $MgCl_2$, 1 mM EGTA (for Mg^{2+}-loaded proteins) or 1 mM $CaCl_2$ (for Ca^{2+}-loaded proteins). The samples were subjected to SDS-PAGE (5% concentrating and 15% resolving gels; 5 μg of protein per lane) and stained with Coomassie Brilliant Blue R-250.
